# The impact of oral health literacy on dental anxiety and utilization of oral health services among dental patients: a cross sectional study

**DOI:** 10.1186/s12903-023-02840-3

**Published:** 2023-03-12

**Authors:** Amira Badran, Khaled Keraa, Mahassen Mohamed Farghaly

**Affiliations:** 1 Faculty of Oral & Dental Medicine, Misr International University, Cairo, Egypt; 2 Faculty of Dentistry, Ain Shams University, Cairo, Egypt

**Keywords:** Oral health literacy, Dental anxiety, Dental health services utilization, ARELAD-30 tool, AMDAS

## Abstract

**Background:**

Low oral health literacy levels and deficient oral health knowledge jeopardize the communication between dentists and patients in different communities. This study aimed to examine the impact and association of oral health literacy with patients’ levels of dental anxiety and their utilization of dental health services.

**Methods:**

This cross-sectional study was conducted at the Misr International University (MIU) dental clinics. The study utilized a structured, interview led questionnaire that was administered by second year dental students, over the period of two successive academic years 2018–2019 and 2019–2020. A total of 440 student interviewed a convenience sample of 440 dental patients: including 269 females (61.1%) and 171 males (38.9%). The questionnaire consisted of four sections; a demographic section, a modified Arabic Rapid Estimate of Adult Literacy (ARELAD-30) Tool that measures the ability of the participants to read 30 commonly used dental terms. This questionnaire was modified by the authors to measure the participants’ knowledge by asking them to choose the most accurate meaning for each word based on their previous knowledge. Scoring was dependent on the participant’s immediate correct pronunciation, as well as comprehension of each word. The Arabic Modified Dental Anxiety Scale (AMDAS) was used to measure the level of dental anxiety, and the dental health service utilization was measured using the Utilization of oral health services questionnaire.

**Results:**

One quarter (24.1%) of the participants read the 30 items of the A-REALD correctly. The average percentage of correct responses to the meaning of the dental terms was 71.2%. There was no statistically significant association between A-REALD and knowledge scores (Spearman’s Correlation coefficient *ρ* = -0.008, *p*-value = 0.872). There was a statistically significant inverse correlation between age and MDAS (Correlation coefficient *ρ* = -0.146, *p*-value = 0.002). A-REALD scores were inversely correlated with time since last visit (Regression coefficient = -0.027, *p*-value = 0.036, with 95% CI: -0.052 – -0.002).

**Conclusion:**

Within the limitations of this study, it can be concluded that oral health literacy is significantly associated to dental health services utilization, while, dental anxiety is related to other variables, such as age and gender.

## Background

The WHO defines health literacy as “the ability to engage with health information and services in a meaningful way” [1]. It involves the tools and actions needed to obtain and understand health information and services necessary to make proper health decisions; and is therefore important for health empowerment [2]. Health literacy is currently recognized as a determinant of health, and is considered a primary cause of health disparities, and has become a public health priority [3, 4]. Comparable to health literacy, oral health literacy (OHL) has also proven to be critical in reducing oral health disparities and in promoting oral health [3]. Limited OHL has been linked to greater risk for oral diseases, poor oral health outcomes, improper oral health behaviors, and reduced utilization of oral health services [5–7].

In spite of the recent technological advancement and the ease of retrieving information regarding oral health via the internet, a survey conducted in the United Kingdom found that one out of every five individuals lacked the fundamental skills required to understand simple information that would help them lead a healthy life [8]. Therefore, there is a need to identify individuals with low OHL in every population and to recognize the factors and determinants related to OHL. This is of great importance in the prevention and control of oral diseases, as some factors which affect the individuals’ oral health such as, the socioeconomic conditions cannot be modified, [2] while OHL, the utilization of oral health services and DA are all modifiable factors that can be improved to serve as a strategy toward the prevention of oral diseases. Accordingly, measuring and identifying the association between these three modifiable variables might be of great help in improving oral health.

Several tools have been developed to measure OHL; most of them focusing on functional literacy; which evaluates the reading and writing skills of a patient. These include abilities such as understanding a prescription or drug dosage, having control of health risk information and using health services [2]. One of the most used tools is the REALD-30, it consists of 30 commonly used dental terms, and has been tested for reliability and validity, and has been translated into several languages. [6]

Dental Anxiety (DA) can be described as the fear related to dental visits and procedures. The prevalence of DA worldwide is high, and ranges from 2 to 30% [9, 10]. It ranks as fourth among common fears and ninth among intense fears [11]. Thus, it is not only considered a mental health issue, but is a public health concern as well [12]. DA usually emerges in pediatric patients and later extends into adulthood [9]. Previous studies have shown that children and women suffer from DA more than adults and men [13].

DA has been related to a number of dental procedures; local anesthesia injection being the most fearful situation, followed by the drilling of teeth, then pain during dental treatment and having instruments in the mouth, respectively [14]. It has been reported to cause 6% of some previously studied populations to avoid utilizing oral health services [15] and may lead to the deterioration of oral health-related quality of life [10, 13]. Some authors have suggested that the improved awareness of relevant risk factors for DA would make it easier for clinicians, especially pediatric dentists to deal with their patients, and would help improve their treatment approaches with anxious patients [16]. Although a previous study related higher levels of DA to the lack of dental health education among a group of students in Yemen [10], yet few studies have investigated the possible link between OHL and DA, focusing primarily on the impact of parents’ OHL on their children’s DA and oral health status [17, 18].

Several scales have been used to evaluate the level of DA. These scales include the dental anxiety scale (DAS), and the modified dental anxiety scale (MDAS), which are the most frequently, used assessment tools in populations worldwide [19].

Utilization of oral health services is represented by the actual attendance of individuals to oral health care facilities [20]. Studies have shown that various factors have impact on individual oral health service utilization [21, 22], including OHL [23], and DA; people with high levels of dental fear visited the dentist less often and demonstrated a longer time between dental visits [24]. Higher DA has also been associated with a greater perceived need for dental treatment, and worse self-rated oral health, and symptom driven visiting patterns [25]. Bouma et al. [26] proposed that anxiety plays a basic role in avoidance behavior, resulting in further negative dental visit experiences. Other studies have indicated that dissatisfaction with health care quality and fees are associated with poor compliance, low utilization, and/ or the termination of treatment [27]. These factors leading to low dental service utilization affect both individuals, and communities, and additionally represent a public health challenge to the nation’s overall oral health [28].

The determinants of OHL and DA, as well as utilization of dental services has been studied extensively; with some findings confirming the link between DA and the utilization of dental services [13, 29], and other conflicting findings regarding the association between dental utilization and OHL [23, 24]. However, the association between OHL and DA has not been investigated sufficiently. Accordingly, the aim of this study was to examine the impact and association of OHL with patients’ levels of DA and their utilization of dental health services.

### Sample size calculation

This study’s power analysis used correlation between OHL and DA as the primary outcome. The effect size ρ = 0.134 was calculated based upon results of a pilot study conducted on 50 subjects from the target population. Using alpha (α) level of (5%) and Beta (β) level of (20%) i.e. power = 80%; the minimum estimated sample size was n = 432 subjects. The sample size calculation was performed using G*Power Version 3.1.9.2.

## Methods

### Study design and study settings

The current, cross sectional study was conducted at the Misr International University (MIU) Dental Clinics Complex. The study utilized a specially structured, interview led questionnaire that was administered by 440 s year dental students of both genders, over the period of two successive academic years 2018–2019 and 2019–2020.

### Ethical approval

The research proposal was approved by the Institutional Review Board at MIU (MIU-IRB-1819-073)) in accordance with the Declaration of Helsinki. The purpose and scope of the study was explained to the participants. Respondents were asked to sign an informed consent form before they were interviewed, and they were informed that their participation in the study was completely voluntary and that they could withdraw at any point of the study.

### Study participants

A total of 440 second year dental students interviewed a convenience sample of 440 dental patients: including 269 females (61.1%) and 171 males (38.9%). The mean age (standard deviation) of all participants was 37.7 (11.3) years old with a minimum of 18 and a maximum of 71 years old. The mean age (standard deviation) of males weas 39.9 (12.6) years old with a minimum of 18 and a maximum of 71 years old while for females it was 36.4 (10.1) years old with a minimum of 19 and a maximum of 63 years old. The level of education was below university level for more than half of the participants. Housewives made up 38.9% of the participants and 46.3% were employed. Most of the participants were married (73.7%). Healthy adults, as well as adults with chronic conditions and those who regularly used medication were included. Subjects with mental or psychological problems and those who were illiterate were excluded from the study because the questionnaire requires the subjects to read a list of dental terms. Completely edentulous patients were also excluded because the questionnaire on utilization of services asked about types of dental services specific for dentate patients. Before conducting the study, orientation sessions and thorough training of the students were performed to ensure that they could conduct the interviews consistently and efficiently. The staff members taught the students to design educational materials; brochures, leaflets and posters that were aimed at improving patients’ knowledge and awareness regarding oral health and the utilization of oral health services. These educational materials were distributed to the enrolled participants after being interviewed, and the students explained and answered any questions or inquiries that the participants had.

### Study instrument

The questionnaire consisted of four sections; a demographic section, a modified Arabic Rapid Estimate of Adult Literacy (ARELAD-30) Tool [6], the Arabic Modified Dental Anxiety Scale (AMDAS) [15], and the Utilization of oral health services questionnaire [27]. A teaching assistant at the Dental Public Health Department carried out a pilot test of the modified questionnaires on a sample of 50 randomly selected patients. The results of this pilot study were not used in the analysis of the study results but were used to modify the study questionnaire in order to improve clarity and understandability.

The demographic section: included questions regarding the patients’ gender, age in years, and educational level which was coded as a 3-level categorical variable. Data about occupation, residence, and marital status were also collected.

Oral health literacy was measured using a validated word recognition test which was a modified version of the Arabic Version of Rapid Estimate of Adult Literacy in Medicine (**A-REALD-30**) [6]. Each participant was given a list of 30 dental terms in Arabic, e.g., enamel, fluoride, orthodontic treatment etc., and asked to read each word aloud to the interviewer. A modification of the questionnaire was made by the authors of this study in which each participant was asked to choose the most accurate meaning for each word based on their previous knowledge. Scoring was dependent on the participant’s immediate correct pronunciation for each word as well as comprehension of each word. The participant received a score of (1) for being able to read the word properly, while pauses, hesitations, and repetitions received a score of (0) [6]. An additional mark was given for being able to choose the correct meaning of the word.

**Fig. 1:**
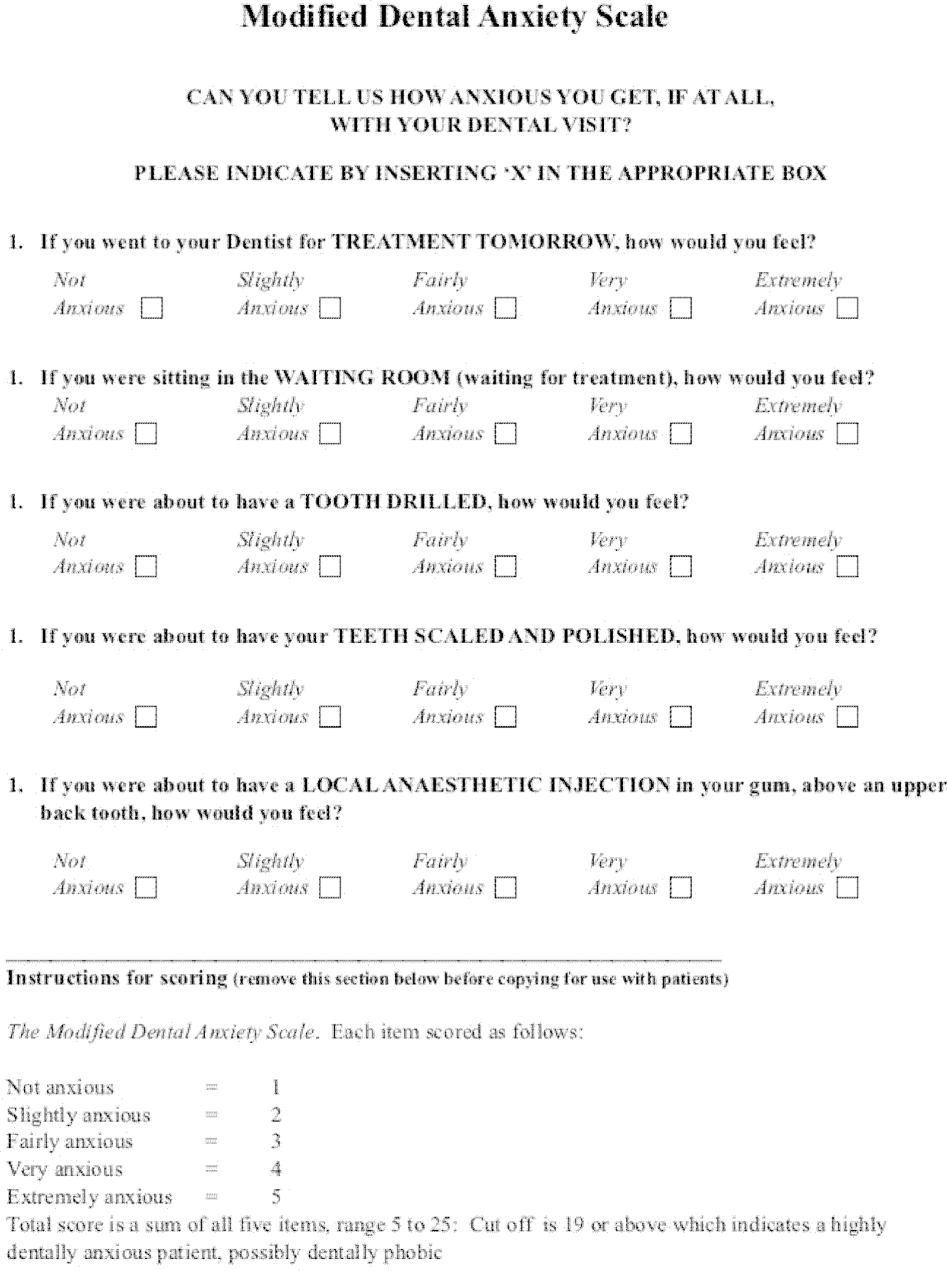
Modified Dental Anxiety Scale Questionnaire

Dental anxiety was measured using the AMDAS. The MDAS (Fig. 1) is a validated questionnaire composed of five questions, each utilizing a five-category Likert rating scale ranging from non-anxious (scored 1) to extremely anxious (scored 5). The total possible score for the scale ranged from 5 to 25. Higher scores represented higher DA. A cutoff point for high DA has been suggested for a score of 19, based on clinical relevance [10]. The questionnaire assesses patients’ anxiety in the following five situations: anticipating a visit to a dental clinic, waiting in the dentist’s office for treatment, sitting on the dental examination chair before drilling of teeth, scaling of teeth, and prior to receiving a local anesthetic injection [15].

Utilization of oral health services was measured using a questionnaire that consisted of six questions measuring patients’ opinion about their last dental visit, type of treatment received, self-evaluation of oral health, and the causes which affected their choice of a dental clinic or hospital for utilizing dental services [27].

### Statistical analysis

Categorical data were presented as frequencies, percentages, and 95% Confidence Interval for the proportions (95% CI). Numerical data were presented as mean and standard deviation (SD) values. The Mann-Whitney U test was used to compare between MDAS scores of males and females. Spearman’s correlation coefficient was used to study the correlations between A-REALD and MDAS, A-REALD and knowledge, and age and MDAS. Linear Regression analysis was used to determine the impact of different variables on DA and the utilization of dental services. Statistical analyses were performed using IBM SPSS Statistics for Windows (version 23.0.; IBM Corp, Armonk, NY).

## Results

This study was conducted on 440 subjects including 269 females (61.1%) and 171 males (38.9%) with a mean age (SD) of 37.7 (11.3) years old. The educational level of 59.4% of the participants was below university level, 33.9% had university education and 6.7% had post-graduate degrees. More than one third of the participants (38.9%) were housewives, 9.1% were unemployed, 5.7% were students, 26.6% had blue collar jobs and 19.7% had white collar jobs. More than half of the participants (53.6%) were from urban areas. The majority of participants (73.7%) were married, and 18.9% were single while 7.3% were divorced or widowed.

The mean (SD) values for the A-REALD were 25.9 (4.5) with a minimum of 3, and a maximum of 30. Approximately one quarter (24.1%) of the participants read the 30 items of the A-REALD correctly.

Results of responses to knowledge about oral health are presented in Table 1. The average percentage of correct responses was 71.2%. The question with the highest percentage of correct responses (97.7%) was related to the transmission of infection if someone uses another person’s tooth brush, while the question with the lowest percentage of correct responses (24.8%) was related to the number of deciduous teeth.

Correlation between A-REALD and knowledge scores: There was no statistically significant association between A-REALD and knowledge scores (Spearman’s Correlation coefficient *ρ* = -0.008, *p*-value = 0.872).

**Fig. 2:**
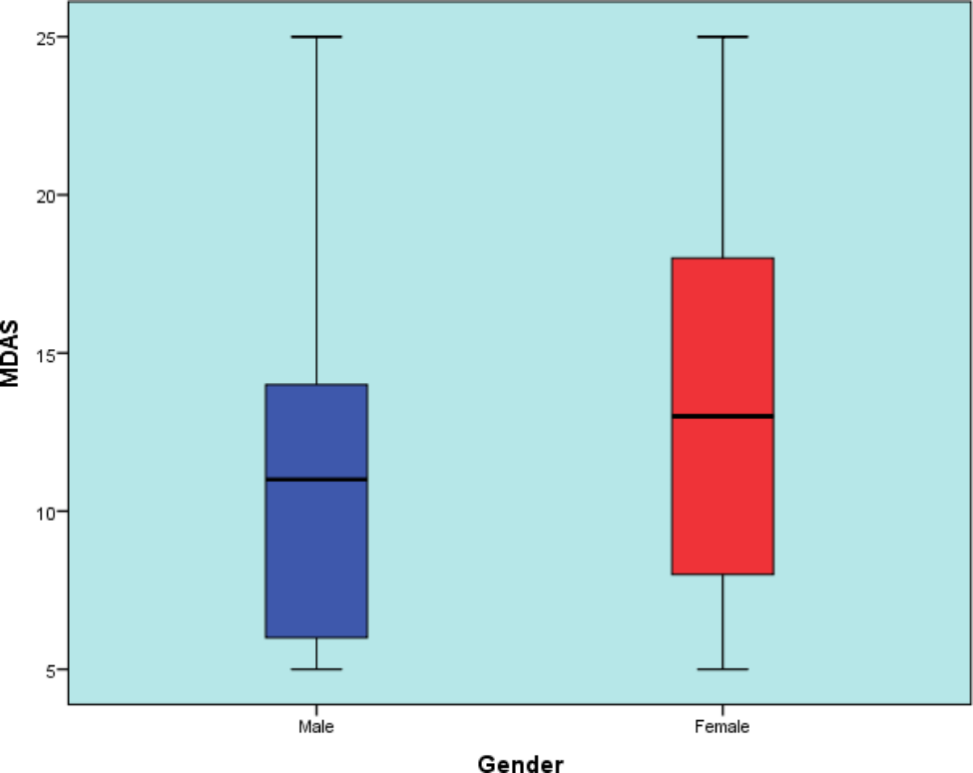
Box plot representing median and range values for MDAS for modified dental anxiety score of males and females

**Fig. 3:**
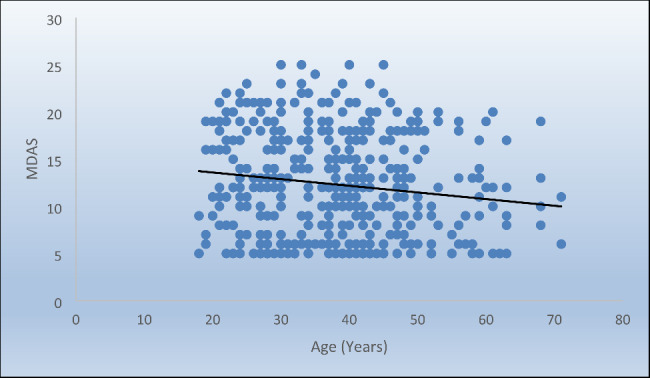
Scatter diagram representing inverse correlation between age and MDAS scores

Responses to MDAS questions are presented in Table 2. The mean (Standard D) values for MDAS was 12.3 (5.4) and ranged from 5 to 25. Since the mean was below 19, the patients were not assessed as anxious. Median and range values for MDAS for modified dental anxiety score of males and females are represented in Fig. 2. The median (range) values for MDAs in males and females were 11 (5–25) and 13 (5–25), respectively. Males showed statistically significantly lower median MDAS scores than females. The correlation between age and MDAS scores are represented in Fig. 3. There was a statistically significant inverse correlation between age and MDAS (Correlation coefficient *ρ* = -0.146, *p*-value = 0.002).

Correlation between A-REALD and MDAS: There was no statistically significant association between A-REALD and MDAS (Correlation coefficient *ρ* = 0.041, *p*-value = 0.391).

Results of responses to utilization of dental services questions are presented in Table 3. More than half of the participants had their last dental visit since less than six months, while almost one fifth of them had their last visit since more than two years. The most visited types of clinic were faculty and private clinics followed by governmental clinics then polyclinics. The most common reason for the previous dental visit was pain in almost one half of the participants followed by treatment, while the least common reason was regular checkups. The most prevalent type of treatment was fillings (26.7%), and prosthodontics (24.6%), while the least prevalent type was implant placement (1%). Almost two thirds of the participants (59.4%) evaluated their oral health as moderate and good, while only (40.7%) reported poor oral health. The most common reason for choosing a dental clinic for receiving treatment was quality of service (62.7%) followed by reputation while the least common cause was suitable appointments availability (21.1%). The most common reason for not choosing a dental clinic was cost (53.2%), followed by fear of infection and inadequate time (30.2% for each cause, respectively), while the least common cause was excessive distance (16.4%).

A linear regression model was constructed to identify significant predictors of DA and the utilization of dental services (Table 4). The dependent variables were MDAS and time since last visit while the independent variables were gender, age, residence, educational level, occupation, social level, and A-REALD score. Regarding DA; gender and age were found to be statistically significant predictors for DA (gender regression coefficient = 1.499, *p*-value = 0.006, with 95% CI: 0.425–2.573) and (Regression coefficient = -0.099, *p*-value < 0.001, with 95% CI: -0.151 – -0.046). Males showed statistically significantly lower median MDAS scores than females while DA was inversely correlated with age.

For utilization of dental services, educational level and A-REALD scores were found to be statistically significant predictors of utilization of dental services. Educational level was inversely correlated with time since last visit; with higher educational levels being associated with less time since last dental visit (Regression coefficient = -0.091, *p*-value = 0.023, with 95% CI: -0.169 – -0.012). A-REALD scores were inversely correlated with time since last visit; with higher A-REALD scores indicating good dental literacy being associated with less time since last dental visit (Regression coefficient = -0.027, *p*-value = 0.036, with 95% CI: -0.052 – -0.002).

**Table 1:** Frequencies (n), percentages (%) and 95% confidence intervals of correct responses to oral health knowledge questions (N = 440)

Oral health knowledge	n	%	95% CI
1. Enamel is the inner layer of teeth.	134	30.5	26.4–34.8
2. Excessive intake of carbonated and acidic beverages lead to erosion of enamel.	310	70.5	66.6–74.8
3. The pulp contains blood vessels that helps nutrition of teeth.	311	70.7	66.6–74.8
4. Untreated pulp inflammation leads to abscess formation.	398	90.5	87.7–93
5. Children teeth are called deciduous and they are 24 teeth.	109	24.8	20.5–28.9
6. Adult teeth are called permanent and they are 32 excluding wisdom teeth.	218	49.5	44.8–54.3
7. Improper use of toothpicks may lead to gingival recession.	355	80.7	77.1–84.3
8. Gingival recession is a main cause of hypersensitivity.	329	74.8	70.5–79.1
9. Sticky sugars e.g. Toffee can cause tooth decay more than fluid sugars like juice.	419	95.2	93.2–97
10. Children who sleep immediately after drinking sugary fluids are more prone to high caries rates.	391	88.9	85.5–91.6
11. Presence of Fluoride in drinking water and toothpastes can cause an increase in bony fractures.	162	36.8	32.3–41.4
12. Fluoride doesn’t protect from tooth decay.	176	40	35.2–44.5
13. Dental abscess is caused by bacteria and leaving it untreated may lead to serious complications.	379	86.1	82.7–89.3
14. Dental abscess could be formed in the gingiva or jaw bones.	356	80.9	77.3–84.8
15. Dental plaque is a sticky substance produced by bacteria on teeth surfaces and bacteria reproduce in it.	302	68.6	64.3–72.7
16. Proper tooth brushing doesn’t help removing plaque.	199	45.2	40.7–49.8
17. Smoking is one cause of halitosis.	403	91.6	88.6–94.1
18. Tooth brushing is one method of preventing halitosis.	420	95.5	93.4–97.3
19. Caries may lead to pulpal inflammation.	426	96.8	95-98.4
20. Sometimes caries is invisible and may lead to tooth fracture.	402	91.4	88.6–93.9
21. Orthodontics is treatment of misaligned teeth.	406	92.3	89.8–94.5
22. Orthodontic treatment in pre-pubertal stage often gives best results.	322	73.2	69.1–77.3
23. Extraction is the best solution to get rid of toothache.	285	64.8	60.2–69.3
24. Early extraction of children teeth is one cause of misaligned teeth.	289	65.7	61.4–70.5
25. Dental floss helps cleaning tight areas in-between teeth.	335	76.1	71.8–80.2
26. It is preferred to use waxed dental floss because it prevents gingival injury.	282	64.1	59.8–68.6
27. Using other person’s toothbrush can transmit infection.	430	97.7	96.1–99.1
28. Hard toothbrushes are better than soft ones.	240	54.5	49.8–59.3
29. Using analgesics without prescription may lead to gastric ulcer.	406	92.3	89.8–94.8
30. Antibiotics can be used to relief pain.	210	47.7	43-52.5

**Table 2:** Percentages of responses to dental anxiety scale questions

Dental anxiety scale		Not anxious	Slightly anxious	Fairly anxious	Very anxious	Extremely anxious
Males (N = 171)	1. If you will go to your dentist for treatment tomorrow, how would you feel?	49.1	25.1	8.8	7.6	9.4
	2. If you were sitting in the waiting room (waiting for treatment), how would you feel?	43.3	22.8	17.5	6.4	9.9
	3. If you were about to have a local anesthetic injection in your gum above an upper back tooth, how would you feel?	37.4	14	18.1	9.4	21.1
	4. If you were about to have a tooth drilled, how would you feel?	38	10.5	15.2	18.7	17.5
	5. If you were about to have your teeth scaled and polished, how would you feel?	64.9	15.8	13.5	1.2	4.7
Females (N = 269)	1. If you will go to your dentist for treatment tomorrow, how would you feel?	32.7	21.2	17.5	16.4	12.3
	2. If you were sitting in the waiting room (waiting for treatment), how would you feel?	30.9	19.3	23	11.9	14.9
	3. If you were about to have a local anesthetic injection in your gum above an upper back tooth, how would you feel?	24.9	16.4	11.5	13.8	33.5
	4. If you were about to have a tooth drilled, how would you feel?	30.5	11.2	14.9	18.6	24.9
	5. If you were about to have your teeth scaled and polished, how would you feel?	52.8	21.9	16	4.8	4.5
Total (N = 440)	1. If you will go to your dentist for treatment tomorrow, how would you feel?	39.1	22.7	14.1	13	11.1
	2. If you were sitting in the waiting room (waiting for treatment), how would you feel?	35.7	20.7	20.9	9.8	13
	3. If you were about to have a local anesthetic injection in your gum above an upper back tooth, how would you feel?	29.8	15.5	14.1	12	28.6
	4. If you were about to have a tooth drilled, how would you feel?	33.3	10.9	15	18.7	22.1
	5. If you were about to have your teeth scaled and polished, how would you feel?	57	19.8	15.2	3.4	4.6

**Table 3:** Frequencies (n) and percentages (%) of responses to utilization of dental services questions

Utilization of dental services	n	%	95% CI
1. Last dental visit:			
a. < 6 months	245	55.7	51.1–60.5
b. 6–12 months	71	16.1	12.7–19.5
c. 1–2 years	47	10.7	8-13.6
d. > 2 years	77	17.5	14.3–21.1
2. Type of clinic:			
a. Governmental	73	16.6	13.4–20
b. Private	165	37.5	33–42
c. Polyclinic	30	6.8	4.8–9.3
d. Faculty	172	39.1	34.5–43.4
3. Reason of last dental visit:			
a. Pain	217	49.3	44.5–54.1
b. Regular check up	32	7.3	5.2–10
c. Treatment:	191	43.4	38.6–48.2
• Filling	51/191	26.7	20.6–33.6
• Root canal treatment	28/191	14.7	10-20.5
• Extraction	38/191	19.9	14.5–26.3
• Prosthodontics	47/191	24.6	18.7–31.3
• Scaling	4/191	2.1	0.6–5.3
• Implant placement	2/191	1	0.1–3.7
4. Self-reported oral health:			
a. Good	72	16.4	13–20
b. Moderate	189	43	38.4–47.5
c. Poor	179	40.7	36.4–45.2
5. Reasons of choosing a dental clinic:			
a. Reputation	222	50.5	45.5–55.2
b. Quality of service	276	62.7	57.7–67
c. Suitable appointments	93	21.1	17.3–24.8
d. Facilities and equipment	189	43	38.4–47.3
e. Friendly staff	178	40.5	36.1–45.2
6. Reasons of not choosing a dental clinic:			
a. Distance	72	16.4	13–20
b. Cost	234	53.2	48.4–58
c. Fear of infection	133	30.2	25.9–34.5
d. Fear of treatment and complications	115	26.1	22-30.5
e. Inadequate time	133	30.2	26.1–34.8

**Table 4:** Results of linear regression analysis model for predictors of dental anxiety and utilization of dental services

Dependent variable	Independent variables	Regression coefficient (*β*)	Standard Error (SE)	*P*-value	95% CI
Dental anxiety (MDAS)	Gender	1.499	0.547	0.006	0.425–2.573
	Age	-0.099	0.027	< 0.001*	-0.151 - -0.046
	Educational level	-0.010	0.180	0.954	-0.364–0.343
	Residence	0.155	0.085	0.715	-0.215–4.257
	Occupation	0.055	0.048	0.341	-0.066–0.994
	Social status	1.090	0.540	0.054	-0.029–2.151
	A-REALD	0.064	0.058	0.264	-0.049–0.178
Time since last visit	Gender	-0.206	0.122	0.092	-0.446–0.034
	Age	-0.004	0.006	0.488	-0.016–0.008
	Educational level	-0.091	0.040	0.023*	-0.169 - -0.012
	Residence	0.047	0.022	0.069	-0.029–1.557
	Occupation	0.177	0.030	0.528	-0.030–0.215
	Social status	-0.104	0.120	0.388	-0.339–0.132
	A-REALD	-0.027	0.013	0.036*	-0.052 – -0.002
	M-DAS	-0.005	0.011	0.643	-0.026–0.016

*: Significant at P ≤ 0.05

## Discussion

Oral health literacy and knowledge of oral health, modulated by the social determinants of health, can generate appropriate oral health decisions, and allow for the formulation of health promotion strategies that can impact individual and community health outcomes [30]. Low OHL and deficient oral health knowledge jeopardizes the communication between health professionals and patients. This increases the need to investigate OHL of different populations and to focus on cultural adaptation of the scientific language so that information can be generated that reaches target populations and generates improved health skills [2].

The results of this study noted a minimum A-REALD score of 3 and a maximum of 30 for the A-REALD scores, with approximately one quarter of the patients being able to read the 30 items of the A-REALD correctly. This would reflect a low level of OHL if only the findings of literacy were considered. Despite this, the scores for oral health knowledge indicated a 71.2% of correct answers which reflect a satisfactory level of knowledge and understanding of dental health terms and conditions. This contrasts with other studies that have suggested that subjects with decreased OHL are likely to have less oral health knowledge when compared with those with higher literacy [4].

This difference highlights the importance of our findings as it reflects the significance of measuring both the ability to read the dental terms and the ability to comprehend the terms as this is more important when it comes to literacy. It also highlights the significance of the patient – dentist relationship and the level of communication between them. Most of the participants in the current study were regular dental patients at the faculty clinics. Accordingly, the difference between the A-REALD scores and knowledge scores could be attributed to the role the dentists who treated those patients played in raising their awareness and knowledge regarding different dental facts and treatment modalities, thus increasing their knowledge scores. Contrary to our findings a study conducted in the United States found an association between low OHL and low oral health services knowledge [31]. Another study reported that individuals with low OHL were significantly more likely to have lower self-efficacy regarding knowledge of how to prevent dental caries and periodontal disease [32].

The literature is rich with studies that demonstrate the factors related to DA and poor oral health outcomes. In the present study, DA rates were higher in females and younger individuals than in males and older age groups. These findings are congruent with previous studies that highlighted age and gender as two important indicators of DA. Although non modifiable, recognizing such factors may help identify patient groups which are predisposed to DA [13, 16, 17]. In the current study, anticipating a local anesthetic injection and tooth drilling were reported as the most fear producing experiences in the dental clinic. This finding is similar to those of other studies which have reported these two procedures as the most fear provoking out of all dental procedures [10, 14]. Previous research has also shown that DA should be considered a public health issue and that several oral symptoms may increase dental fear, especially when patients expect potentially threatening interventions [12].

Managing dental anxiety represents a real challenge for dentists, especially pediatric dentists. Psychotherapeutic interventions, pharmacological interventions, or a combination of both had been suggested based on the level of anxiety, the characteristics of the patient, and other clinical considerations. Cognitive behavior therapy is currently the most accepted psychological treatment for anxiety. In circumstances, where the patient requires surgical interventions, or refuses psychotherapeutic interventions, or is considered dental-phobic, pharmacological therapies such as sedation or general anesthesia should be sought. [10, 33]

Regarding the correlation between OHL and DA, there was no statistically significant association between A-REALD and MDAS. Similarly, the regression model used in the current study revealed no impact of A-REALD scores on the MDAS scores. These contrasts with two previous studies that found that parents’ high levels of DA were significantly associated with a low degree of OHL [17, 18]. However, the difference between our findings and these two previous studies might be related to the fact that they only measured the REALD as an indicator of literacy and did not account for knowledge scores that might differ from the literacy scores. Additionally, these two studies were relating OHL to DA of the participants’ children not to the respondents themselves which might skew the results. Additionally, the significant multivariate association of the MDAS scores with other variables, such as oral health, income, age, and gender; that may affect the results [18]. This was confirmed by our findings which indicated that both the gender and age were significant predictors of DA. Informing clinicians about these findings would likely help them to respond to the treatment needs of patients with low OHL and to consider the effective communication and the other factors that may impact their level of DA.

This study found the utilization of dental services to be satisfactory with more than half of the participants having their last dental visit within the previous six months. However, this data should be interpreted cautiously as the study participants were already dental patients seeking dental services. Different results might be encountered in different settings. Pain followed by continuation of dental treatment was the most common reason for seeking dental services. This is like a study that concluded that without pain, many patients viewed oral health-care services as elective [34].

Restorative treatments were the most prevalent dental treatment type, while implants were the least prevalent treatment. Faculty and private clinics were preferred over governmental and polyclinics. Almost two thirds of the patients evaluated their oral health as being moderate or good. This is an important finding, as self- perceived oral health is considered a useful subjective measure of a person’s oral health, and accordingly, is an important indicator of dental needs within a population [5].

Investigating patients’ motivating factors to utilize dental services may also provide useful information which can improve understanding of patient behavior and their opinions about the dental services. Quality of dental care and a good reputation were found to be the most important factors in seeking and encouraging continuous utilization of services in a chosen clinic. In contrast, high costs and fear of infection were found to be the primary discouraging factors to continue utilization of dental services in dental clinics. The distance to the dental clinic was the least discouraging factor to utilization of dental services in our study. This contrasted with other studies which found that dental clinics which were far from home was the main discouraging factor to continue utilization of dental services. The authors of these studies related these findings to the effects of crowded streets and jammed traffics [27, 34].

Regarding the predictors and impact of OHL on the utilization of dental services; the current study showed that the educational level of an individual is a significant predictor for dental service utilization and this is in accordance with a study in which the logistic regression revealed that patients who had a higher education level were twice as likely to be regular in their oral health care visits [20]. The OHL level based on the A-REALD scores was also found to be a significant predictor for the utilization of dental services. Additionally, previous studies have suggested that low OHL leads to a decreased adherence to positive oral health behaviors [23].

A study which did not find an association between OHL and the utilization of dental services explained this lack of association as a possible reflection of ineffective doctor-patient communication [24]. This would confirm the interpretation of our findings and highlights the role of the dentists in raising awareness and improving patients’ knowledge of oral health.

## Limitations

The findings of the present study should be interpreted, considering the following limitations. Firstly, the data were collected from a convenience sample of patients from a university-based dental clinic. Accordingly, this study reflects only the OHL and dental behaviors of patients attending a university-based dental clinic only and does not necessarily reflect that of the greater community. Secondly, the collected data relied on self-perceived outcomes, which could be biased as the patients may over or underestimate their responses. Another important limitation is related to the fact that the data collection for this study has ended before the emergence of COVID 19, knowing that the pandemic had influenced the psychological status of dental patients [35], the levels of dental anxiety and fear of transmission of infections from dental clinics might have changed during, and post the pandemic crisis. Accordingly, it is advisable to compare the pre and post pandemic DA levels among dental patients. Finally, the cross-sectional design of this study prevents it from identifying causality. Association was noted only between dental utilization and OHL. Further longitudinal studies or clinical trials may be required to extend the findings reported in the present study.

## Conclusion

Within the study limitations, it can be concluded that OHL is a significant predictor of dental health services utilization, while, DA is associated with variables other than OHL, such as age and gender.

## Data Availability

The datasets used and/or analyzed during the current study are not publicly available due to the regulations and rules of the university that prohibit the availability of data on public repository. However, the data will be available from the corresponding author on reasonable request.

## References

[1] World Health Organization (1998). Health promotion glossary.

[2] Batista M, Lawrence H, Sousa M (2018). Oral health literacy and oral health outcomes in an adult population in Brazil. BMC Public Health.

[3] Baskaradoss J (2018). Relationship between oral health literacy and oral health status. BMC Oral Health.

[4] Firmino R, Martins C, Faria L, Paiva S, Garcia A, Fraiz F, Ferreira F (2018). Association of oral health literacy with oral health behaviors, perception, knowledge, and dental treatment related outcomes: a systematic review and meta-analysis. J Public Health Dent.

[5] Tenani C, Checchi M, Bado F, Ju X, Jamieson L, Mialhe F (2020). Influence of oral health literacy on dissatisfaction with oral health among older people. Gerodontology.

[6] Tadakamadla S, Quadri M, Pakpour A, Zailai A, Sayed M, Mashyakhy M, Inamdar A, Tadakamadla J (2014). Reliability and validity of Arabic Rapid Estimate of adult literacy in Dentistry (AREALD-30) in Saudi Arabia. BMC Oral Health.

[7] Ismail A, Ardini Y, Mohamad N, Bakar H. Association between parental oral health literacy and children’s oral health status. Revista Latinoamericana de Hipertensión. Vol. 13 - Nº 3, 2018

[8] Council NC. Health Literacy: being able to make the most of health.London: 2004. Available at: http://www.cdc.gov/healthliteracy/statedata/index.html

[9] Grisolia B, Santos A, Dhyppolito I, Buchanan H, Hill K, Oliveira B (2020). Prevalence of dental anxiety in children and adolescents globally: a systematic review with meta-analyses. Int J Paediatr Dent.

[10] Appukuttan D (2016). Strategies to manage patients with dental anxiety and dental phobia: literature review. Clin Cosmet Invest Dentistry.

[11] Nascimento DL, Araújo AC, Gusmão ES, Cimões R (2011). Anxiety, and fear of dental treatment among users of public health services. Oral Health Prev Dent.

[12] Crego A, Díaz M, Armfied J, Romero M (2014). From public mental health to community oral health: the impact of dental anxiety and fear on dental status. Front Public Health.

[13] Yakar B, Kaygusuz T, Pirincci E (2019). Evaluation of Dental anxiety and fear in patients who admitted to the Faculty of Dentistry: which patients are more risky in terms of Dental anxiety. Ethiop J Health Sci.

[14] Madfa A, Al-Zubaidi S, Shibam A, Al-ansi W, Beshari L, Al-Haj A, AL-Jawfi K. Dental Anxiety and Fear among a University Population in a Sample from Yemen. Preprint. DOI: 10.21203/rs.2.21375/v1.

[15] Bahamma M, Hassan M (2014). Validity, and reliability of an arabic version of the modified dental anxiety scale in saudi adults. Saudi Med J.

[16] Goh E, Beech N, Johnson N (2020). Dental anxiety in adult patients treated by dental students: a systematic review. J Dent Educ.

[17] Barasuol J, Assunção L, Fraiz F, Menezes J (2017). Oral health literacy as a predictor of Dental anxiety in parents of children undergoing Dental Treatment. J Dent Child.

[18] Shin W, Braun T, Inglehart M, Habil P (2014). Parents’ dental anxiety and oral health literacy: effects on parents’ and children’s oral health-related experiences. J Public Health Dent.

[19] Fayad M, Elbieh A, Baig M, Alruwaili S (2017). Prevalence of Dental anxiety among Dental Patients in Saudi Arabia. J Int Soc Prev Community Dentistry.

[20] Quadri F, Jafari F, Albeshri A, Zailai A (2018). Factors influencing patients’ utilization of Dental Health Services in Jazan, Kingdom of Saudi Arabia. Int J Clin Pediatr Dentistry.

[21] Al-Jaber. Da’ar OB. Primary health care centers, extent of challenges and demand for oral health care in Riyadh, Saudi Arabia.BMC Health Serv Res. 2016Nov; 16(1):628. 10.1186/s12913-016-1876-6 PMC5096290 27809919

[22] Okoro CA, Strine T, Eke PI, Dhingra SS, Balluz LS. The association between depression and anxiety and use of oral health services and tooth loss. Community Dent Oral Epidemiol. 2012 Apr;40(2):134–44. 10.1111/j.1600-0528.2011.00637.x 21883356

[23] Firmino R, Martins C, Faria L, Paiva S, Granville-Garcia A, Fraiz F, Ferreira F (2018). Association of oral health literacy with oral health behaviors, perception, knowledge, and dental treatment related outcomes: a systematic review and meta-analysis. J Public Health Dent.

[24] Burgette J, Lee J, Baker A, Vann W. Is Dental Utilization Associated with Oral Health Literacy?Journal of Dental Research95(2):160–166. DOI: 10.1177/0022034515617457 10.1177/0022034515617457 PMC4720957 26567035

[25] Armfield J, Stewart J, Spencer A. The vicious cycle of dental fear: exploring the interplay between oral health, service utilization and dental fear. BMC Oral Health. 2007;7(1). 10.1186/1472-6831-7-1. 10.1186/1472-6831-7-1 PMC1784087 17222356

[26] Bouma J, Uitenbroek D, Westert G, van de Schaub RM (1987). Pathways to full mouth extraction. Community Dent Oral Epidemiol.

[27] Al Johara A. Al-Hussyeen.Factors affecting utilization of dental health services and satisfaction among adolescent females in Riyadh City. Saudi Dent J. 2010;22:19–25. 10.1016/j.sdentj.2009.12.004. 10.1016/j.sdentj.2009.12.004 PMC3723370 23960475

[28] Petersen PE. World Health Organization global policy for improvement of oral health – World Health Assembly 2007.Int Dent J 2008Jun; 58(3):115–121. 10.1111/j.1875-595x.2008.tb00185.x 18630105

[29] Sohn W, Ismail AI (2005). Regular dental visits and dental anxiety in an adult dentate population. J Am Dent Assoc.

[30] Nutbeam D (2000). Health literacy as a public health goal: a challenge for a contemporary health education and communication strategies into the 21st century. Health Promot Int.

[31] Macek MD, Atchison KA, Wells W, Haynes D, Parker RM, Chen H. Did you know Medicare does not usually include a dental benefit? Findings from a multi-site investigation of oral health literacy. J Public Health Dent. 2017Mar;77(2):95–8. 10.1111/jphd.12199 PMC5557019 28079917

[32] Macek MD, Atchison KA, Chen H, Wells W, Haynes D, Parker RM, Azzo S. Oral health conceptual knowledge and its relationship with oral health outcomes: findings from a multi-site health literacy study. Commun Dent Oral Epidemiol. 2017 Aug;45(4):323–9. 10.1111/cdoe.12294 PMC5498245 28271537

[33] Pourabbas R, Ghahramani N, Sadighi M, Pournaghi Azar F, Ghojazadeh M (2022). Effect of conscious sedation use on anxiety reduction, and patient and surgeon satisfaction in dental implant surgeries: a systematic review and meta-analysis. Dent Med Probl.

[34] Bommireddy V, Pachava S, Viswanath V, Talluri D, Ravoori S, Sanikommu S (2017). Oral Health Care seeking behaviors, and influencing factors among south indian rural adults: A Cross sectional study. J Indian Association Public Health Dentistry.

[35] Daltaban Ö, Aytekin Z (2022). Fear and anxiety of COVID-19 in dental patients during the COVID‐19 pandemic: a cross-sectional survey in Turkey. Dent Med Probl.

